# The Radiologic Evaluation of Pediatric Acute Abdomen; Results of Tertiary Referral Center

**DOI:** 10.5334/jbr-btr.883

**Published:** 2015-12-30

**Authors:** Güliz Yılmaz, Gökhan Pekindil, Süha Akpınar, Aydın Şencan, Cüneyt Günşar, Erol Mir, Mine Özkol

**Affiliations:** 1 Department of Radiology, Faculty of Medicine, Near East University, Nicosia, North Cyprus, Turkey; 2 Department of Radiology, Faculty of Medicine, Celal Bayar University, Manisa, Turkey; 3 Department of Pediatric Surgery, Faculty of Medicine, Celal Bayar University, Manisa, Turkey

**Keywords:** plain abdominal radiographs, ultrasonography, computed tomography, pediatric acute abdomen

## Abstract

**Purpose:** In this study we aimed to evaluate the radiological examinations of the pediatric patients who were operated with initial diagnosis of acute abdomen.

**Methods:** We retrospectively reviewed the clinical records and imaging findings of 252 children. All patients were evaluated by plain abdominal radiographs (PAX) and ultrasonography (US). Only 10 patients were examined using computed tomography (CT). The findings of the PAX, US and CT of each patient were determined from their detailed archive records according to their clinical diagnosis.

**Results:** The most frequent pathology was appendicitis in our study whereas the other pathologies were invagination, ovarian torsion, the complications of Meckel’s diverticulum, gastrointestinal obstruction and tuboovarian abscess in decreasing frequency. PAXs were valuable in diagnosis of the patients with ileus. It has been showed that US was the most useful for patients with appendicitis and invagination. CT was performed only in 4% of our cases as an advanced diagnostic method.

**Conclusion:** The pediatric patients with acute abdomen have been evaluated radiologically by PAX and US routinely and frequently. CT was performed as an advanced diagnostic method very rarely. CT would be utilized to a lower extent as a more advanced method of imaging in unsolved patient group, as US and PAX solve the pediatric acute abdominal pathologies in high percentages.

## Background

Abdominal pain in children is a frequent symptom among referrals to the pediatric emergency departments. Besides anamnesis and physical examination, radiological examination is also required for the differential diagnosis of abdominal pain. The majority of abdominal pain in children is because of internal cause, and is generally not associated with intra-abdominal solid organs [1,2]. The most frequent internal cause of abdominal pain is gastroenteritis, and the most frequent surgical cause is appendicitis [3].

## Objectives

The purpose of this study was to re-evaluate the radiological findings in pediatric cases undergoing surgery with the prediagnosis of acute abdomen.

## Materials, Patients and Methods

This study included 252 cases which were suspected of having acute abdomen with clinical and laboratory findings, and had undergone surgery. The patients’ radiological examination results and post-operative diagnoses were retrospectively compared. It was noted that all cases with prediagnosis of acute abdomen concurrently had undergone plain abdominal radiographs (PAX) and ultrasonography (US). Plain abdominal radiographs were used to determine the presence of air-fluid level, abdominal distention due to gas, dilated bowel loops, opasity in the lower-right quadrant (appendicolitis), and calcified pelvic lesion. The air-fluid level was accepted as the presence of more than two air-fluid levels of 2.5–3 cm. Intestinal dilation was defined as bowel loops of more than 3 cm in width, and appendocolitis as an opasity adjacent to the cecum in the right-lower quadrant. Following PAX, the patients underwent abdominal US examination, which was performed by radiology assistants on emergency call. The US protocol of acute abdomen in our clinic includes visualization of the appendix, compressibility of the appendix, free fluid in abdomen, lymph nodes, invagination (target sign), structure and vascularization of the ovaries, dilated bowel loops, and intra-abdominal mass. The normal appendix (appendicitis: (–) on US) was defined as a blind-ending tubular structure arising from the cecum, which could be compressed with the probe, and had an antero-posterior (AP) diameter of less than 6 mm. The examination result was accepted to be within normal limits when the appendix could not be visualized and accompanying secondary findings were absent. The pathological appendix on US was defined as a non-compressible blind-ending tubular structure arising from cecum, which had an AP diameter of more than 6 mm, and showed no peristaltism. Following the US examination, cases suspected of ovarian pathology were further examined for the vascularization of both ovaries, using the color Doppler ultrasonography (CDUS).

Ten cases which could not be diagnosed with PAX or US were further examined with whole abdomen computed tomography (CT), with oral and intravenous contrast material administration. The CT images in the digital data system were retrospectively studied.

## Results

Of the patients, 88 (35%) were girls, 164 (65%) were boys, and their ages ranged from one to seventeen (with a mean age of 8.9 years). Among cases operated for acute abdomen, the most frequent cause was appendicitis, which was found in 218 (86%) of the cases. The other pathologies identified, in the order of frequency, were invagination (7%), ovarian torsion (3%), Meckel’s diverticulum (2%), intestinal obstruction (1.5%), and tubo-ovarian abscess (0.5%). The PAX findings in different pathologies associated with acute abdomen have been presented in Table 1, and the US results of all cases, and of cases with acute appendicitis, are presented in Table 2 and Table 3.

**Table 1:** Findings on plain abdominal radiographs (PAX) in acute abdomen cases.

	Level	N	Gas dist	Level + Apcolit	Level + dilated loops	Apcolit	Total

Acute abdomen	103	99	31	9	6	4	252
Acute appendicitis	87	89	29	9	0	4	218
Invagination	12	1	1	0	3	0	17
Ovarian torsion	0	6	1	0	0	0	7
Meckel’s diverticulum	1	3	0	0	1	0	5
Intestinal obstruction	2	0	0	0	2	0	4
Tubo-ovarian abscess	1	0	0	0	0	0	1

**N:** Normal, **Gas dist:** Gas distention, **Apcolit:** Appendicolitis, **Level:** Air-fluid level, **Level + apcolit:** Air-fluid level + appendicolitis, **Level + dilated loops:** Air-fluid level + dilated intestinal loops.

**Table 2:** Ultrasound imaging findings in all acute abdomen cases.^*^

	Ap (+)	Fluid	Lymph node	Heter. fat tissue	Ap	N	Inv (+)	Inv (–)	Enlarged Ovary	Ovar per	Dil. loop	Mass

**A.ap (218)**	159	106	77	64	36	59						
**Inv (17)**		8	9				13	4				
**OT (7)**	1	3							5	3		
**MD (5)**	1	3	2			1						
**IO(4)**						2					2	
**TOA (1)**		1										1

**A.ap:** Acute appendicitis, **Inv:** Invagination, **OT:** Ovarian torsion, **MD:** Complications of Meckel’s diverticulum, **IO:** Intestinal obstruction, **TOA:** Tubo-ovarian abscess, **Ap (+):** Detection of pathologic apendicitis **Heter. fat tissue:** Heterogeneity in periappendicular fat tissue, **Ap:** Appendicolitis **N:** Normal, **Inv (+):** Detection of invaginated intestinal segment with USI, **Inv (–):** No detection of invaginated intestinal segment with USI, **Ovar per:** Hypoperfusion or no perfusion of blood through ovary, **Dil. loop:** Dilated intestinal loop.

*Numbers within parentheses show the number of cases.

**Table 3:** Detailed ultrasound imaging (USI) findings in acute appendicitis cases.

USI finding	Number of cases

Enlarged appendix	159 (73%)
Appendix (–)	59 (27%)
Periappendicular fluid	106 (49%)
Lymph node in right lower quadrant	77 (35%)
Appendicolitis	36 (16%)
Heterogeneity in periappendicular fat tissue	64 (29%)

Of the 218 cases post-operatively diagnosed as appendicitis, 59 (27%) had given false-negative results on the pre-operative US examination, and among these, 15 (7%) had yielded normal US findings. Of the 15 cases with normal US findings, 14 were intraoperatively diagnosed as acute, non-perforated appendicitis, and one as perforated appendicitis. The remaining 44 cases (20%), accepted as appendicitis-negative for showing no dilated appendix, but had other US findings such as free fluid, lymph nodes, etc. However, among the cases accepted as appendicitis-positive on the US examination, two cases were later found to be a false-positive, with the diagnosis of Meckel’s diverticulitis and ovarian torsion (the AP diameter of appendix was 6.2 mm in the case of Meckel’s diverticulitis and 6.5 mm in the case of ovarian torsion).

Among ten cases also examined with CT, there were findings supporting acute appendicitis in three, associated complications of Meckel’s diverticulum in three, ovarian torsion in two, invagination in one and tubo-ovarian abscess in one cases. The CT findings of ten acute abdomen cases are shown in Table 4.

**Table 4:** Computed tomography (CT) imaging findings in all acute abdomen cases.

	Apcolit	Level	Fluid	Lymph Node	Target Sign	TBW	Dilated loops	Mass	Heter. Fat Tissue

**A.ap (3)**	2	2	1				2		
**Inv (1)**			1	1	1	1			
**OT (2)**								2	2
**MD (3)**			1			1	1	1	1
**TOA (1)**			1					1	1

**A.ap:** Acute Appendicitis, **Inv:** Invagination**, OT:** Ovarian Torsion, **MD:** Meckel’s Diverticulum, **TOA:** Tuba-ovarian abscess, **Apcolit:** Appendicolitis, **Level:** Air-fluid level, **dilated loops:** dilated intestinal loops, **TBW:** thickened bowel wall, **Heter. Fat Tissue:** heterogenity of fatty tissue.

## Discussion

Acute abdominal pain, which is a frequent complaint in pediatric patients, is an important issue in pediatric emergency due to having both medical and surgical causes in its etiology. Acute appendicitis is the cause of abdominal pain that most frequently needs surgical intervention [1,2,3].

Although PAX is preferred as the first line for the diagnosis of children being referred with acute abdominal pain, its sensitivity is low and its contribution to the diagnosis is non-specific—except in cases suspected to have intestinal obstruction or perforation [4,5]. Sixty-one percent of all cases in our study contained abnormal findings determined by PAX. This high rate may be due to the large number of cases being referred to our tertiary center at a late stage of acute abdomen with abdominal obstruction. Air-fluid level (41%), which was the most common finding on PAX in our study, was detected in invagination (70%) and intestinal obstruction (50%) with high rates compared to other abdominal pathologies.

US is widely used in pediatric patients, because it is practical, has no radiation, and is non-invasive. Appendicitis, inflammatory bowel disease, invagination, and cystic or solid masses were the pathologies mainly diagnosed by US in our series [3,6,7]. In our study group 75% of all our cases were diagnosed by US because of the high rate of acute appendicitis and invagination. The most common findings were enlarged appendix and invaginated bowel loop as expected. With the widespread usage of US, differentiation of acute appendicitis from the other acute abdominal pathologies had improved [8]. The ratio of negative laporatomy has reduced with usage of US [9]. Besides, minor causes of acute abdomen such as intraabdominal free fluid, heterogenity of fatty tissue, and mesenteric lymph nodes could be detected by US. The disadvantages of US are its operator dependency and limited diagnostic value in cases of obesity, overlying gas, or perforation [7].

CT is performed rarely in pediatric cases preferably in complicated situations or in cases where US is not applicable, particularly in case of obesity, because it has the disadvantages of high radiation and need for contrast material [3].

Appendicitis was the most common pathology with a prevelence of 86% in our study. It has been reported that PAX provides positive radiological findings in 50–84% of acute appendicitis cases [10]. In our series, PAX detected pathology in 59% of the cases. The PAX findings which were not specific for appendicitis were air-fluid level (40%), gas distention (13%), air-fluid level + appendicolitis (4%), and appendicolitis (2%).

**Figure 1:**
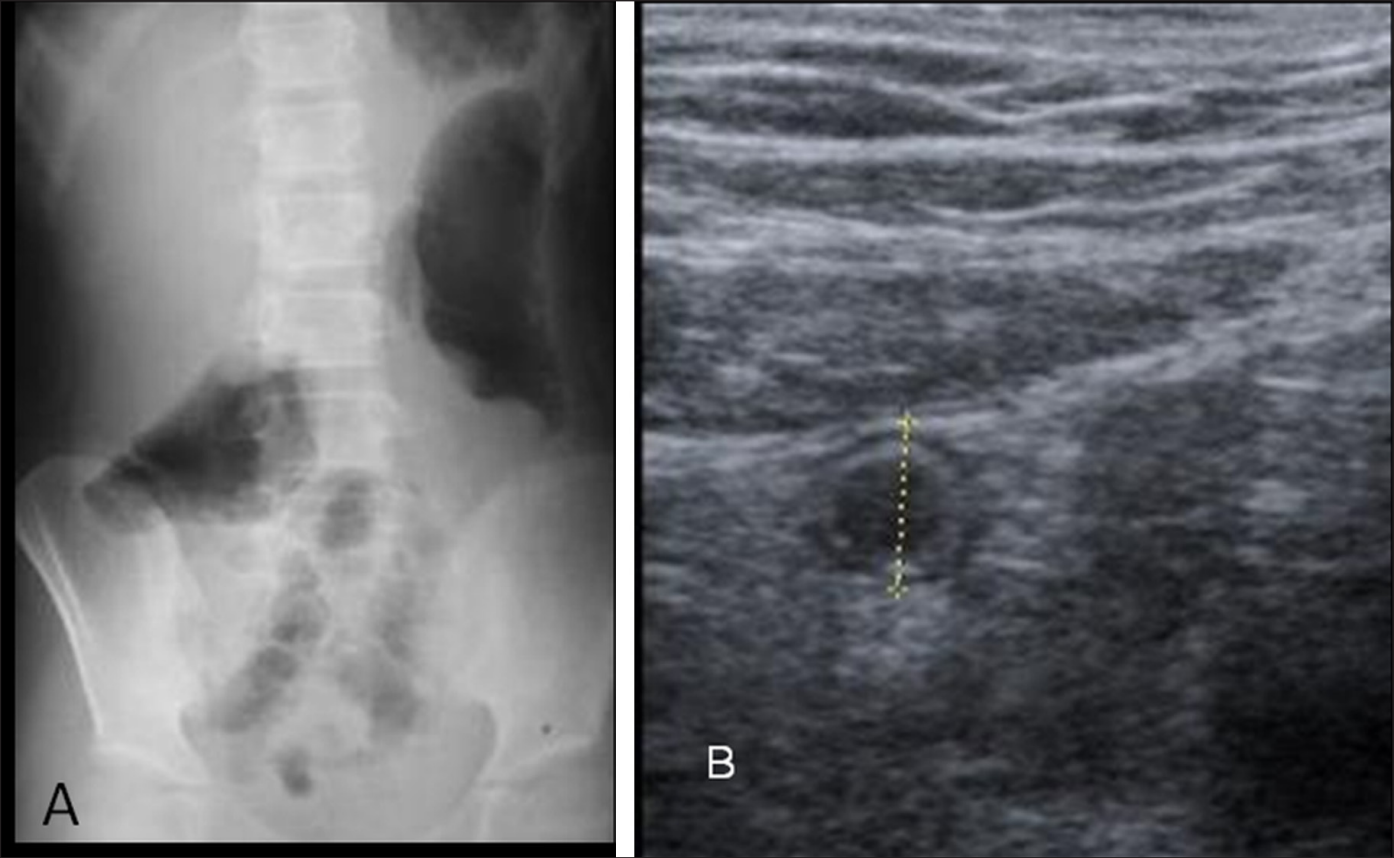
A nine-year-old patient presenting with complaints of abdominal pain localized in the right-lower abdominal quadrant, nausea, and vomiting was examined by PAX (Figure 1A) and US examination (Figure 1B). The patient underwent surgery with the prediagnosis of acute appendicitis and the definitive diagnosis was acute appendicitis. **(A)** Distended colon with gas observed with use of PAX. **(B)** Abdominal US shows the antero-posterior diameter of the non-compressible appendix at 7.3 mm.

The most frequently detected pathology, and the only specific US imaging finding, of the acute appendicitis cases was an enlarged appendix, which has a diameter of over 6 mm (Figure 1). The rate of detection of an enlarged appendix in such cases has been reported as 80–84% in the literature, whereas it was lower (73%) than the literature in our series [11,12]. This could be due to the performance of US assistants who had a different emergency US experience. Furthermore, among acute appendicitis cases diagnosed by pathology, the AP diameter of the appendix was measured 4.8 mm in one case and 5–5.9 mm in 12 cases by US. This finding is significant when non-compressibility of the appendix is considered with the AP diameter under 6 mm.

Periappendicular fluid and pericecal echogenicity are reported in appendicitis cases with the incidence of 5–50% and 16–64% in the literature [6,12]. In our series, the higher prevalence of periappendicular fluid in perforated appendicitis (73%) may be because of the time elapsed patients reach to our tertiary hospital. The prevalence of increased pericecal echogenicity was higher in perforated (40%) and plastron-complicated (90%) appendicitis cases.

The image of appendicolitis is reliable proof of acute appendicitis that is detected as round or oval echogenicities on US and hyperdensities on CT, which is more sensitive than PAX [13,14]. In our series, its prevalence was 16% in and 67% in CT.

CT is performed in cases with negative US examination findings, whereas clinical suspicion is persistant for acute appendicitis in emergency situations [15]. The ratio of negative appendectomy and perforation are decreased in cases utilizing CT examination. Although CT is the most sensitive and specific (94–99%) modality for the diagnosis of acute appendicitis in children, it is rarely used because of its disadvantages [16]. As mentioned before, only three patients were diagnosed by CT in our study.

Invagination, which was the second most frequent pathology in our patients, is more common in males. Compareable with the literature, 76% of our invagination cases were males; 47% of which, were children under the age of one and 65% of which were children under age two [17,18].

**Figure 2:**
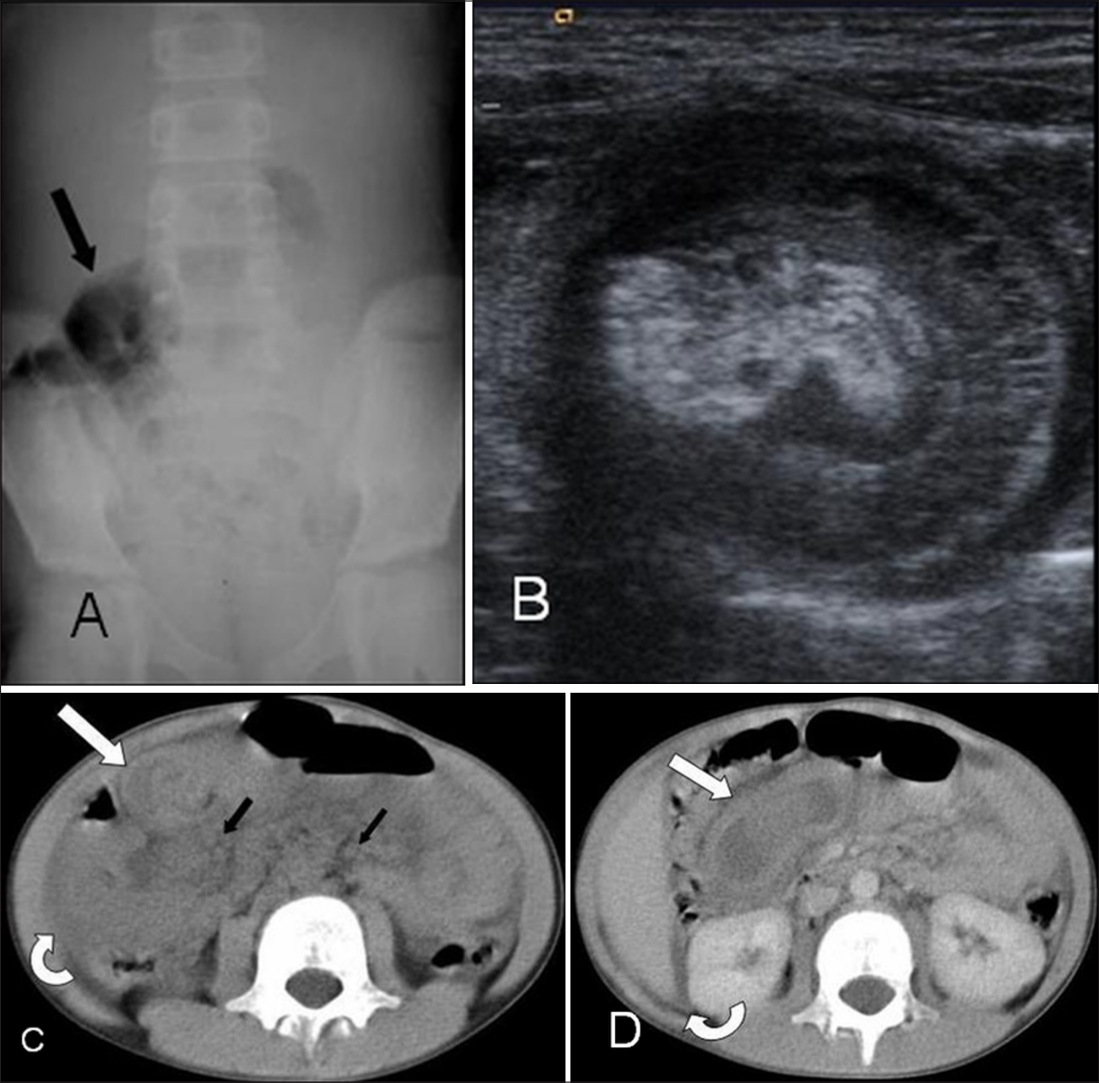
A nine-year-old boy with Henoch-Schoeinlein purpura presenting with abdominal pain, who received the post-operative diagnosis of ileoileal invagination. **(A)** PAX examination shows no marked radiopathological finding except bowel distention with gas in the right-lower abdominal quadrant, indicated by black arrow. **(B)** The zoomed ultrasound image shows the target sign which is consistent with intestinal invagination is present. CT without contrast **(C)** and with contrast **(D)** show the level of invagination. Invaginated intestinal segments and thickening in intestinal walls—thought to be ileoileal invagination—formed the target sign (white arrow). Furthermore, paraaortic lymph nodes adjacent to the invagination (black arrow) and free fluid around the liver and intestinal loops (curved arrow) were observed.

The PAX results were within normal limits in 6% of our invagination patients, whereas this was in 35% in the series of Çalışkan et al. [18]. The presence of air-fluid level was detected in 70% of our patients while Çalışkan et al. reported an incidence of 65% with PAX [18]. US is the most valuable non-invasive diagnostic method that is performed to assess the presence of an invaginated bowel segment, mesenterium, and lymph nodes as a target sign [18,19]. The *target sign*, a finding specific for invagination, was detected in 76% of our patients with US, consistent with the literature. Normally, CT is not indicated for the diagnosis of invagination, but is performed if a dragging sign is considered (for example, lymphoma) [19,20]. Invaginated bowel segments as ileoileal target sign and thickened bowel walls, along with mesenteric lymph nodes were observed on CT, which was performed to determine the level of invagination in a case with Henoch-Schoenlein purpura (Figure 2).

Ovarian torsion occurs in all age groups including the neonatal period, but on average, it most frequently occurs at age 10, as in our cases [21,22]. All ovarian torsions occured in the right ovary—as was was reported in two-thirds of the cases—because the left ovary is immobilized in a tight space near the sigmoid colon.

Apparently, there is no specific finding for overian torsion on PAX. It was stated in the literature that only the calcification of ovarian teratoma could be noticed [23,24]. However, no pathological finding was reported in our seven cases which had undergone PAX examination.

**Figure 3:**
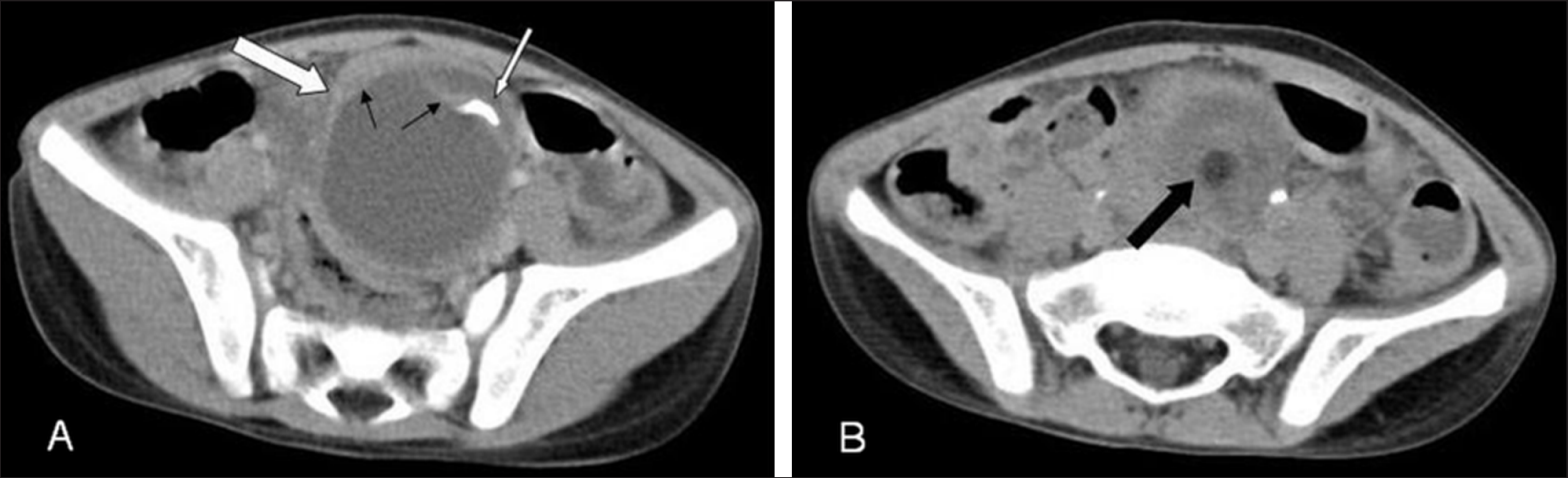
Non-contrast **(A)** and contrast enhanced **(B)** computed tomography (CT) scans show a calcified (white thin arrow), encompasses an area of fat density (thick black arrow) and septa (thin black arrow) 6 cm-sized smooth-walled cystic mass (white thick arrow) distorting the surrounding structures, consistent with teratoma of a 5-year-old girl in the pelvic region. The post-operative diagnosis of the case was right ovarian teratoma and ovarian torsion.

Although the torsioned ovary is observed as markedly enlarged and edematous on US, the diagnosis is established by the absence of arterial blood flow in the ovarian parenchyma [25]. In 40% of necrotized torsioned ovaries, high-resistant or low-speed peripheral arterial flow can be detected with CDUS, but no central venous flow can be observed. However, in cases where necrosis has not developed, both peripheral and central arterial-venous flow forms can be observed [26]. We determined reduced or no arterial flow in the torsioned ovaries with concurrently performed CDUS examination in 43% of our cases, compatible with the literature. US has had a significant role in the diagnosis of ovarian torsion though it is not the definitive diagnostic tool [24]. In our study, abdominal CT which was performed in two cases (29%) with ovarian torsion after detecting ovarian masses showed teratomas with calcifications and fat density in the masses and heterogenity in the surrounding fatty tissue, but CT provided no data of the arterial blood flow in the ovaries (Figure 3). Although US is quite a valuable primary imaging technique in the case of pelvic masses or pain in children, CT is useful in the staging and follow-up of tumors that may be present [27].

Meckel’s diverticulum, the fourth in the order of frequency in our study group, becomes symptomatic under age two in 60% of the cases [28]. In our series, 40% of the cases operated for complications of Meckel’s diverticulum were under age two. The reported complications of Meckel’s diverticulum are bleeding (32–40%), obstruction (invagination or volvulus) (35%) and diverticulitis (11–22%) [29]. In our cases, the most frequent complication was diverticulitis (60%), followed by volvulus (20%) and perforation (20%). The complications of Meckel’s diverticulum can be assessed with US in case of negative scintigraphy findings or atypical clinical findings and symptoms [29]. In our study group, Meckel’s diverticulum could not be detected on US, whereas secondary imaging findings such as fluid and lymph nodes were noticed.

Meckel’s diverticulitis is observed as a blind-ending tubular, round or oval structure surrounded by inflammation on CT. Bennett et al. performed CT on 55% of cases of diverticulitis and could not detect diverticulitis in 20% of these patients because of findings of obstruction that did not permit the use of oral contrast material [30]. Likewise, the CT imagings of our cases showed secondary findings related to complications, but no diverticulitis.

All of our intestinal obstruction cases were caused by post-operative adhesions. The results of PAX in patients pre-diagnosed as intestinal obstruction have been reported to be diagnostic in 50–60%, suspicious in 20–30%, and normal, nonspecific or false in 10–20% of the cases [31]. In our series, air-fluid level and dilated bowel loops were detected in 50% of cases by using PAX. Plain abdominal radiography, which is the initial examination technique in obstruction due to its wide availability and relatively low cost, is used as a basis for triage for further imaging work-up and assist in the therapeutic decision [32].

US is not frequently used for the diagnosis of obstruction because of the gas distention in the intestines and also adhesions, which are the the most frequent cause of obstruction [33]. CT shows a high sensitivity, specificity and reliability in the diagnosis of small bowel obstruction—particularly in children of age over two—that is still not widely used in the diagnosis of obstruction in children [34].

**Figure 4:**
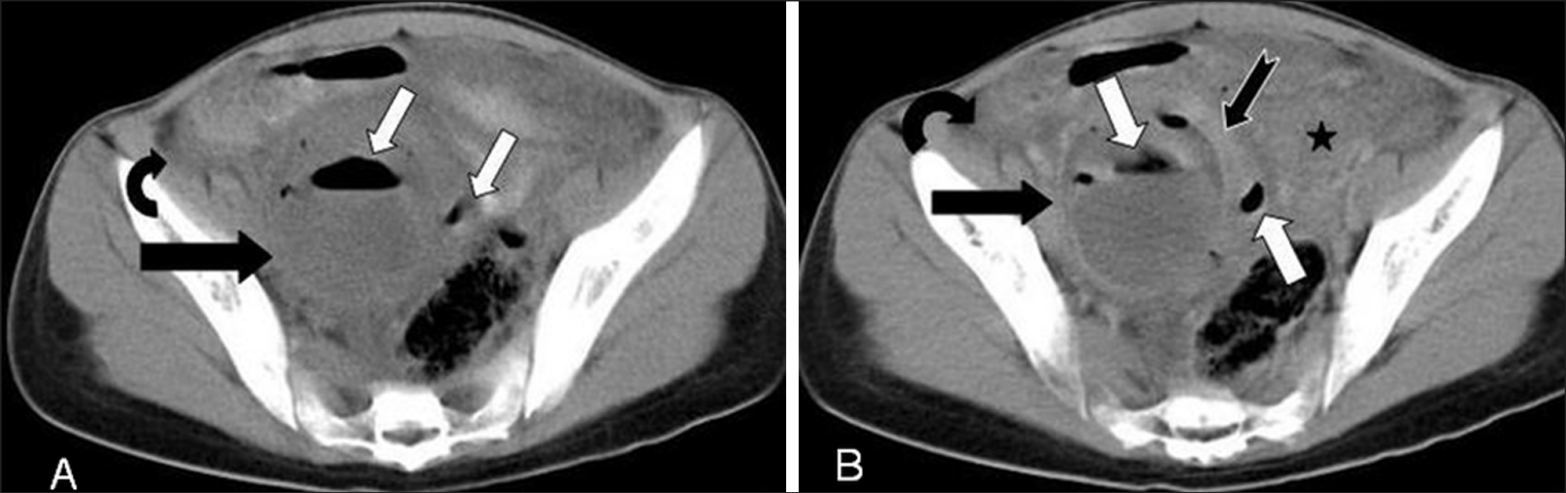
A 14-year-old patient with abdominal pain in right-lower quadrant, who had a history of appendectomy was examined by abdominal CT. Non-contrast **(A)** and contrast enhanced CT **(B)** revealed a 7 cm mass (black arrow) in the right adnexal region with air densities (white arrow) and a peripheral rim-type and contrast-uptake of the wall (black jagged arrow), consistent with tubo-ovarian abscess. In the pelvic region, free fluid (curved arrow) around the bowel loops and heterogeneity (star) in the surrounding fat tissue were also observed. The post-operative diagnosis of the case was right tubo-ovarian abscess.

In the PAX examination of cases with pelvic mass, soft tissue mass and adynamic ileus may be detected [35]. In our case with tubo-ovarian abscess, PAX showed an air-fluid level as a finding of ileus and US showed an intra-abdominal mass and free fluid. CT is complementary to US in cases of tubo-ovarian abscess. Although non-specific, the most frequent finding of tubo-ovarian abscess is a thick-walled mass of fluid-density with internal septations localized in the adnexal region on CT [35]. The CT findings in our case with tubo-ovarian abcess were compatible with the literature (Figure 4).

Finally, according to our study PAX is more beneficial for the diagnosis of invagination and intestinal obstruction whereas acute appendicitis and invagination are diagnosed by US with the highest ratios (73% and 76%), respectively. It is possible that PAX could be the initial examination method in children with the symptoms of obstruction and US might be performed especially in acute appendicitis or invagination cases without additional radiation doses. CT would be utilized to a lower extent as a more advanced method of imaging in unsolved patient group, as US and PAX solve the pediatric acute abdominal pathologies in high percentages.

**Figure 5:**
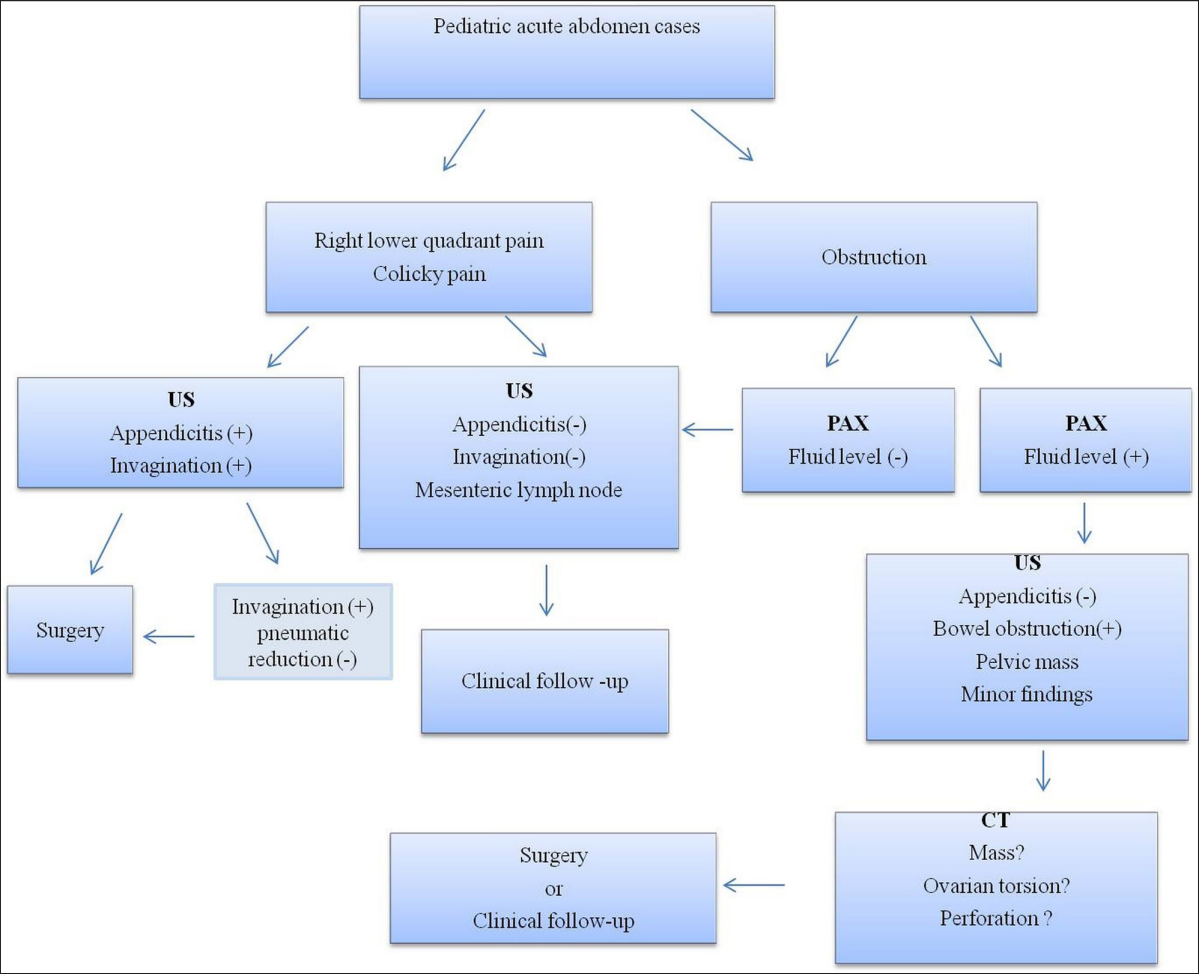
Practical algorithm for pediatric acute abdomen cases.

In conclusion, a practical algorithm could be suggested in order to identify pediatric acute abdomen cases. US would be the first diagnostic method in pediatric populations with right lower quadrant or colicky pain, no additional examination is needed for detecting acute appendicitis or invagination. On the other hand, if there is a pelvic mass with internal calcification then further examination will require CT. If there is a clinical suspicion of obstruction, PAX will be the initial diagnostic technique. However, in the case of insufficient PAX findings to explain underlying pathology, and if additional sonographic finding suggesting bowel obstruction such as dilatation, wall thickening of bowel, and free fluid or pelvic mass, CT might be an additional diagnostic tool (Figure 5).

## Competing Interests

The authors declare that they have no competing interests.
